# Broken Piece of Silicone Suction Catheter in Upper Alimentary Tract of a Neonate

**Published:** 2010-08-14

**Authors:** Bilal Mirza, Muhammad Saleem, Afzal Sheikh

**Affiliations:** Department of Pediatric Surgery, The Children's Hospital and the Institute of Child Health Lahore, Pakistan

**Keywords:** Esophageal foreign body, Neonate, Laryngoscopy

## Abstract

Esophageal foreign bodies (FB) are common in adults and children. These are rarely reported in infants and neonates. A 2-day-old newborn was referred to our hospital with history of accidental intrusion of soft silicone suction catheter into the upper gastrointestinal tract (GIT). X-ray chest and abdomen confirmed the presence of suction tube in esophagus and stomach. The suction catheter was retrieved successfully at direct laryngoscopy.

## INTRODUCTION

Esophageal foreign bodies are commonly encountered in adults and children [1 , 2]. The common esophageal foreign bodies in children are coins, beads, fish bone etc. Small and smooth gastro-esophageal FBs that can pass through the pylorus may be observed for spontaneous passage through anus. However, large / sharp FBs may need active intervention to avoid their symptoms and complications [2 , 3].

Esophageal FBs in infants are rare; rarer still is their occurrence in newborn. Only four cases of neonatal GIT foreign bodies have been reported in Pubmed [1]. We report probably youngest of all the neonates with esophageal foreign body.

## CASE REPORT

A two-day-old male baby was referred to our institution with complaints of vomiting after every feed since birth. The referral letter revealed that the baby was born through spontaneous vaginal delivery in a private hospital; and during newborn resuscitation, the silicone suction catheter was accidentally detached from the suction machine piping and baby swallowed it. Since then the patient started vomiting after every feed. The baby was vitally stable. A radiograph of chest and upper abdomen showed a tube curled up in stomach and esophagus. Patient was taken to the operation theatre and with direct laryngoscopy under general anesthesia a soft silicon suction catheter was retrieved from the upper esophagus (Fig. 1 , 2). Patient showed uneventful recovery and was symptoms free at 3 months follow up.

**Figure:**
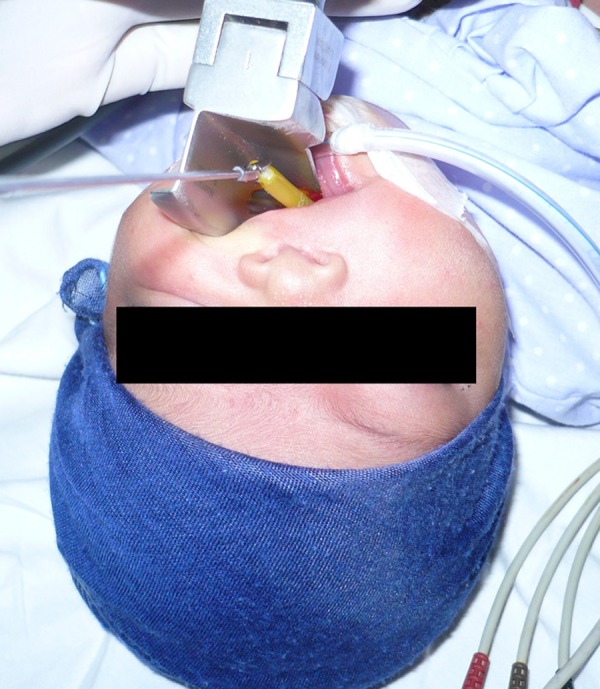
Figure 1: Retrieval of the FB with direct laryngoscopy

**Figure:**
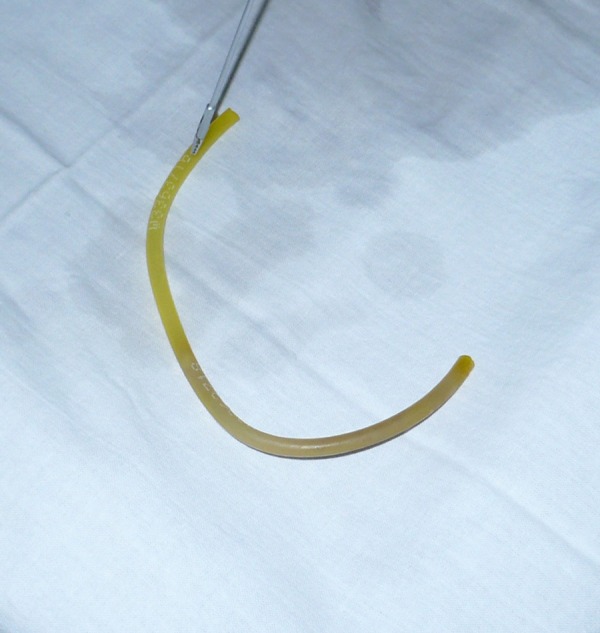
Figure 2: Silicone suction catheter after retrieval

## DISCUSSION

Esophageal foreign bodies are rarely encountered in neonates. After that age, when the milestone of grasping and putting everything in mouth are achieved, the incidence of FB in GIT starts rising. About 80% of all pediatric GIT foreign bodies occur between 6 months and 3 years of age. Zameer et al extensively reviewed the literature regarding GIT foreign bodies in neonates. They were able to find only three cases [1]. 

The usual symptoms of a FB in esophagus are nausea, vomiting, dysphagia, respiratory difficulty, neck pain and hemetemesis. The symptoms usually depend upon the type, size and nature of the FB. Esophageal FB can, sometimes, create fatal complications such as esophageal perforation, subcutaneous emphysema, retro-esophageal abscess, pneumothorax and pneumomediastinum, esophago-aortic fistula, mediastinitis and lung abscess [1 , 3 4 , 5 , 6]. 

There are three types of esophageal FBs. Small esophageal FBs such as beads, buttons, rubber pieces, and button batteries etc; large FBs such as impacted bolus of meat, coins, ornaments and fish bone; and lastly the long FB such as piece of wood, long piece of bone and bezoars. The reported soft FBs are pieces of rubber, rubber-eraser, rubber pallets and buttons, pieces of plastic bags, paper, pieces of clothes, meat, surgical gauze and sponges [1 , 4 , 6 , 7 , 8]. 

Most of small FBs do not need any active management as about 80% of all FBs pass spontaneously from GIT [4]. Large and long FBs usually need intervention for their retrieval [4 , 5 , 6]. It is estimated that only 10-20% of all GIT foreign bodies require endoscopic retrieval, whereas, only 1% require surgical exploration [1]. The management options for esophageal FBs are; retrieval by direct laryngoscopy; esophagoscopy and by open method [1 , 4 , 7 , 8]. Small foreign bodies usually at the level of cricopharyngeus are removed by direct laryngoscopy. The examples are coin, beads and fish bone retrieval. The foreign bodies below this level usually require esophagoscopy or open surgical procedures for their removal [1 , 2 , 4 , 6]. 

Our case is unique because of unusual age of presentation, the mode of intrusion and the FB residing in stomach and esophagus that was retrieved successfully with direct laryngoscopy. It is inferred that casual newborn resuscitation carries a number of life threatening risks for the patient. It may end up in iatrogenic-FBs in the alimentary tract of newborn as happened in this case. Moreover, early referral to a specialized tertiary centre and prompt management can avoid many complications.

## Footnotes

**Source of Support:** Nil

**Conflict of Interest:** None declared
